# Association of PET/CT-Derived Metabolic Parameters with Pathological Complete Response After Neoadjuvant Systemic Therapy in Breast Cancer: An Analysis Across Molecular Subtypes

**DOI:** 10.3390/diagnostics16182909

**Published:** 2026-09-09

**Authors:** Zeliha Birsin, Mehmet Taner Bodur, Selin Cebeci, Seda Jeral Evinç, Ebru Çiçek, Nebi Serkan Demirci, Onur Erdem Şahin, Özkan Alan

**Affiliations:** 1 Department of Medical Oncology, Cerrahpaşa Faculty of Medicine, Istanbul University-Cerrahpaşa, Istanbul 34098, Türkiye; 2 Department of Nuclear Medicine, Cerrahpaşa Faculty of Medicine, Istanbul University-Cerrahpaşa, Istanbul 34098, Türkiye

**Keywords:** breast cancer, neoadjuvant systemic therapy, pathological complete response, ^18^F-FDG PET/CT, ΔSUVmax, metabolic response, molecular subtype

## Abstract

**Background**: Pathological complete response (pCR) following neoadjuvant systemic therapy (NST) is associated with improved outcomes in breast cancer. We evaluated the association between PET/CT-derived metabolic parameters and pCR and explored whether these associations differed according to molecular subtype. **Methods**: This retrospective study included 162 patients with stage II–III breast cancer who received NST followed by surgery and underwent ^18^F-FDG PET/CT at baseline and after completion of NST before surgery. Baseline SUVmax, post-treatment SUVmax, and percentage change in SUVmax (ΔSUVmax) were assessed for the primary tumor and axillary lymph nodes. Multivariable logistic regression and model performance analyses were performed. **Results**: Sixty-three patients (38.9%) achieved pCR. Higher ΔPrimary Tumor SUVmax and lower post-treatment primary tumor SUVmax were independently associated with pCR in separate multivariable models. The corresponding model AUCs were 0.837 and 0.848. Addition of PET-derived parameters to clinical-only models increased the AUC by 0.101 and 0.132, respectively (DeLong *p* = 0.001 and *p* < 0.001). No significant interaction was observed between ΔPrimary Tumor SUVmax and molecular subtype; in exploratory subgroup analyses, the association reached statistical significance in the HER2-positive subgroup only. **Conclusions**: PET/CT-derived metabolic parameters were independently associated with pCR and provided additional discriminatory information beyond clinicopathological factors. No significant interaction with molecular subtype was observed.

## 1. Introduction

Breast cancer is the most frequently diagnosed malignancy among women worldwide and remains a major cause of cancer-related mortality [1]. Neoadjuvant systemic therapy (NST) has become a standard treatment approach for patients with locally advanced and biologically aggressive disease, allowing for tumor downstaging and in vivo assessment of treatment sensitivity [2,3]. Pathological complete response (pCR) following NST has been consistently associated with improved long-term outcomes, particularly in HER2-positive and triple-negative breast cancer (TNBC), and is widely accepted as a clinically meaningful surrogate endpoint [4].

Accurate prediction of pCR remains an important clinical objective. Conventional imaging modalities primarily evaluate anatomical changes; however, residual masses detected after treatment may represent either viable tumor tissue or treatment-related fibrosis, limiting the accuracy of morphological assessment alone. In contrast, ^18^F-fluorodeoxyglucose positron emission tomography/computed tomography (^18^F-FDG PET/CT) enables the evaluation of tumor metabolic activity and may identify treatment response before measurable anatomical changes occur [5,6]. Several studies have demonstrated an association between PET-derived metabolic parameters and pCR following NST [7,8,9]. More recently, PET-guided treatment de-escalation and escalation strategies, particularly in HER2-positive breast cancer, have highlighted the growing clinical relevance of metabolic response assessment [10,11].

Although PET/CT-derived metabolic response has been widely investigated as a predictor of pCR, several uncertainties remain. Previous studies have evaluated baseline, post-treatment, and treatment-induced changes in metabolic parameters, with inconsistent findings regarding their relative predictive value, likely reflecting differences in patient populations, molecular subtypes, treatment regimens, imaging timing, and PET-derived metrics [5,9,12,13]. As PET-guided treatment strategies continue to evolve, further validation of PET/CT-derived metabolic parameters in contemporary real-world patient populations remains clinically relevant.

Therefore, we retrospectively evaluated the association between baseline, post-treatment, and treatment-induced changes in PET/CT-derived metabolic parameters and pCR in patients with breast cancer treated with NST. We assessed metabolic responses in both the primary tumor and axillary lymph nodes and further explored the association between primary tumor metabolic response and pCR across different molecular subtypes.

## 2. Materials and Methods

### 2.1. Study Design and Patient Inclusion Criteria

This retrospective study included patients with pathologically confirmed breast cancer who were diagnosed between January 2015 and March 2024. Eligible patients had non-metastatic breast cancer and were considered suitable candidates for NST following multidisciplinary tumor board evaluation. Patients were required to have undergone both baseline and post-treatment ^18^F-FDG PET/CT examinations, with the post-treatment PET/CT performed after completion of NST and before surgery.

Molecular subtypes were determined according to the American Society of Clinical Oncology/College of American Pathologists (ASCO/CAP) guidelines. Hormone receptor positivity was defined as estrogen receptor (ER) and/or progesterone receptor (PR) expression in ≥1% of tumor cells. HER2 positivity was defined as an immunohistochemistry (IHC) score of 3+ or an IHC score of 2+ with HER2 gene amplification confirmed by fluorescence in situ hybridization (FISH). Considering the sample size, patients were classified into three molecular subtype groups: HR+/HER2−, HER2-positive, and TNBC.

Patients were excluded if they had metastatic disease at diagnosis, did not complete NST, did not undergo surgery, were younger than 18 years of age, had an Eastern Cooperative Oncology Group (ECOG) performance status >2, had a history of another active malignancy, or had incomplete clinical, imaging, or pathological data.

### 2.2. Neoadjuvant Treatment and Surgical Management

Neoadjuvant treatment regimens were selected according to tumor biology, drug availability, and contemporary clinical practice guidelines. Patients with HER2-positive disease received anthracycline-based or non-anthracycline-based chemotherapy combined with either single-agent or dual anti-HER2 therapy. Patients with TNBC received anthracycline- and taxane-based chemotherapy, with the addition of platinum agents according to patient characteristics and multidisciplinary tumor board recommendations, and immunotherapy when available and clinically indicated, while patients with HR+/HER2− disease received anthracycline- and taxane-based neoadjuvant chemotherapy according to institutional practice.

Following completion of NST, all patients underwent definitive breast surgery, including either breast-conserving surgery or mastectomy. Axillary surgical management consisted of sentinel lymph node biopsy (SLNB), axillary lymph node dissection (ALND), or both, according to clinical nodal status, treatment response, and institutional guidelines.

### 2.3. Data Collection, Study Variables and Outcome Definitions

Clinical, pathological, treatment-related, and imaging data were retrieved from archived medical records and institutional electronic databases. Collected variables included age, menopausal status, ECOG performance status, histological subtype, tumor grade, Ki-67 proliferation index, clinical T and N stages, molecular subtype, neoadjuvant treatment characteristics, and surgical procedures.

The primary imaging variables included baseline, post-treatment, and treatment-induced changes in PET/CT-derived metabolic parameters of the primary tumor and axillary lymph nodes.

The primary study endpoint was pCR, defined as the absence of residual invasive disease in the breast and axillary lymph nodes following NST (ypT0/is ypN0).

### 2.4. PET/CT Acquisition and Image Analysis

Both baseline and post-treatment PET/CT examinations were required for study inclusion. Patients fasted for at least 4 h before each PET study, and blood glucose levels were confirmed to be below 160 mg/dL prior to tracer administration. Post-treatment PET/CT examinations were performed 2–4 weeks after completion of NST and before surgery. An intravenous dose of 3.7–4.44 MBq/kg of ^18^F-FDG was administered. Image acquisitions were performed using a Discovery 710 PET/CT scanner (GE Healthcare, Milwaukee, WI, USA). PET images were acquired from the vertex to the mid-thigh.

All images were reviewed by a single experienced nuclear medicine physician who was aware of the diagnosis of breast cancer but was blinded to pathological response and axillary pathological status. For the primary tumor, SUVmax was measured using a volume of interest (VOI) placed over the most metabolically active portion of the lesion. For axillary assessment, PET-positive nodal involvement was defined as the presence of at least one lymph node demonstrating FDG uptake clearly greater than that of surrounding tissues and contralateral axillary lymph nodes. SUVmax was measured from the most metabolically active axillary lymph node using a VOI. When post-treatment lymph nodes were too small for reliable VOI delineation, measurements were obtained using a manually drawn region of interest (ROI).

SUVmax values of the primary tumor and axillary lymph nodes were recorded at baseline and after neoadjuvant treatment and analyzed as quantitative PET-derived parameters. The percentage change in SUVmax (ΔSUVmax) was calculated as follows:

ΔSUVmax (%) = [(SUVmaxbaseline − SUVmaxpost-treatment)/SUVmaxbaseline] × 100

**Figure 1:**
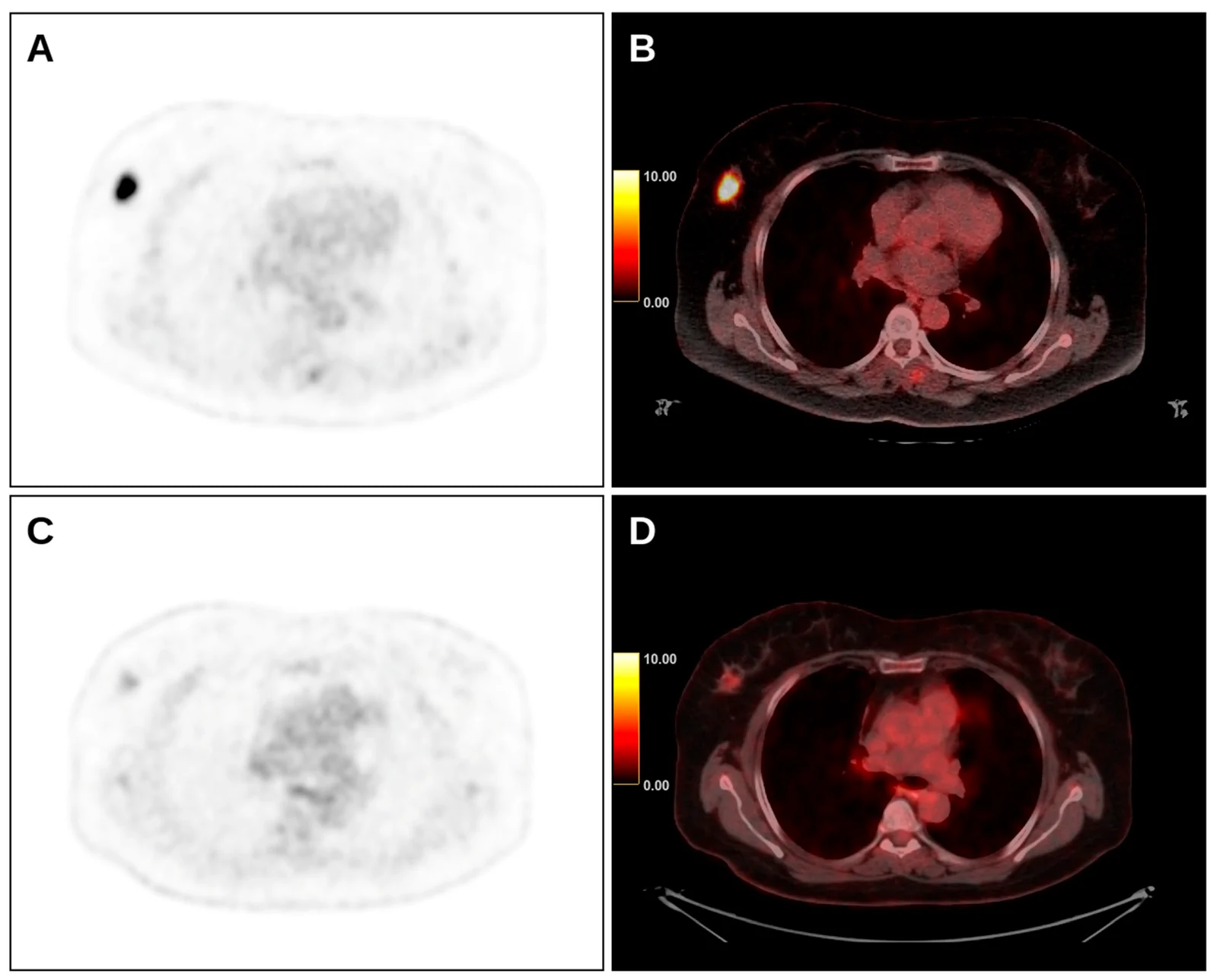
Representative PET/CT images of a patient who achieved a pCR following NST. Baseline PET (**A**) and fused PET/CT (**B**) images demonstrate an irregular right breast mass with intense FDG uptake. Post-treatment PET (**C**) and fused PET/CT (**D**) images show mild residual focal FDG uptake within the tumor bed, which was interpreted as residual metabolic activity. However, pathological examination revealed a complete pathological response despite the presence of mild residual FDG uptake. The calculated ΔSUVmax was 82%.

Representative PET/CT images illustrating metabolic response assessment using ΔSUVmax are shown in Figure 1.

For the primary tumor, baseline SUVmax, post-treatment SUVmax and ΔPrimary Tumor SUVmax were recorded. For axillary lymph nodes, baseline SUVmax, post-treatment SUVmax and ΔAxillary SUVmax were evaluated. Patients with biopsy-proven axillary metastases but no visually identifiable FDG-avid axillary lymph node on baseline PET/CT were included in the overall cohort; however, post-treatment axillary SUVmax and ΔAxillary SUVmax analyses were performed only in patients with PET-positive axillary disease at baseline.

### 2.5. Statistical Analysis

Statistical analyses were performed using SPSS software (version 26.0; IBM Corp., Armonk, NY, USA) and R software (version 4.6.1; R Core Team, R Foundation for Statistical Computing, Vienna, Austria) (https://www.r-project.org/ (accessed on 26 August 2026)). Continuous variables were assessed for normality using the Shapiro–Wilk test and visual inspection methods. As none of the continuous variables showed a normal distribution, they were summarized as median (range). Categorical variables were presented as frequencies and percentages. Comparisons between groups were performed using the Mann–Whitney U test for continuous variables and the Chi-square test or Fisher’s exact test for categorical variables, as appropriate.

Univariable logistic regression analyses were performed to identify factors associated with pCR. Variables with a *p* value <0.10 in univariable analyses were considered for inclusion in the multivariable logistic regression models. Given the mathematical relationship between baseline, post-treatment, and ΔSUVmax parameters, mathematically coupled PET variables were not entered simultaneously into the same multivariable model. Accordingly, two separate multivariable models were constructed. Model 1 included ΔSUVmax parameters together with eligible clinicopathological covariates, whereas Model 2 included baseline and post-treatment SUVmax parameters together with the corresponding clinicopathological covariates. The results were reported as odds ratios (ORs) with 95% confidence intervals (CIs).

Receiver operating characteristic (ROC) curve analyses were performed for individual PET/CT-derived metabolic parameters to evaluate their discriminatory ability for pCR. Areas under the ROC curves (AUCs) with 95% CIs were calculated, and cut-off values were identified using the Youden index.

The discriminatory performance of the multivariable logistic regression models was evaluated separately. For each model, predicted probabilities of pCR were obtained from the fitted logistic regression model and used to construct ROC curves and calculate AUCs with 95% CIs. Precision–recall analysis was performed using the model-predicted probabilities, and the area under the precision–recall curve (AUPRC) was calculated for each model. Model calibration was assessed by comparing predicted and observed pCR probabilities using calibration plots. Internal validation was performed using 1000 bootstrap resamples, and optimism-corrected AUCs were calculated.

To assess the incremental value of PET-derived parameters beyond clinicopathological factors, clinical-only models including tumor grade, clinical T stage, and molecular subtype were compared with the corresponding PET-augmented models. Differences in AUC between the clinical-only and PET-augmented models were assessed using DeLong’s test for correlated ROC curves. Nested clinical-only and PET-augmented models were additionally compared using likelihood-ratio tests.

Subgroup and interaction analyses were performed according to molecular subtype. All statistical tests were two-sided, and a *p* value <0.05 was considered statistically significant.

### 2.6. Use of Generative Artificial Intelligence

**Figure 2:**
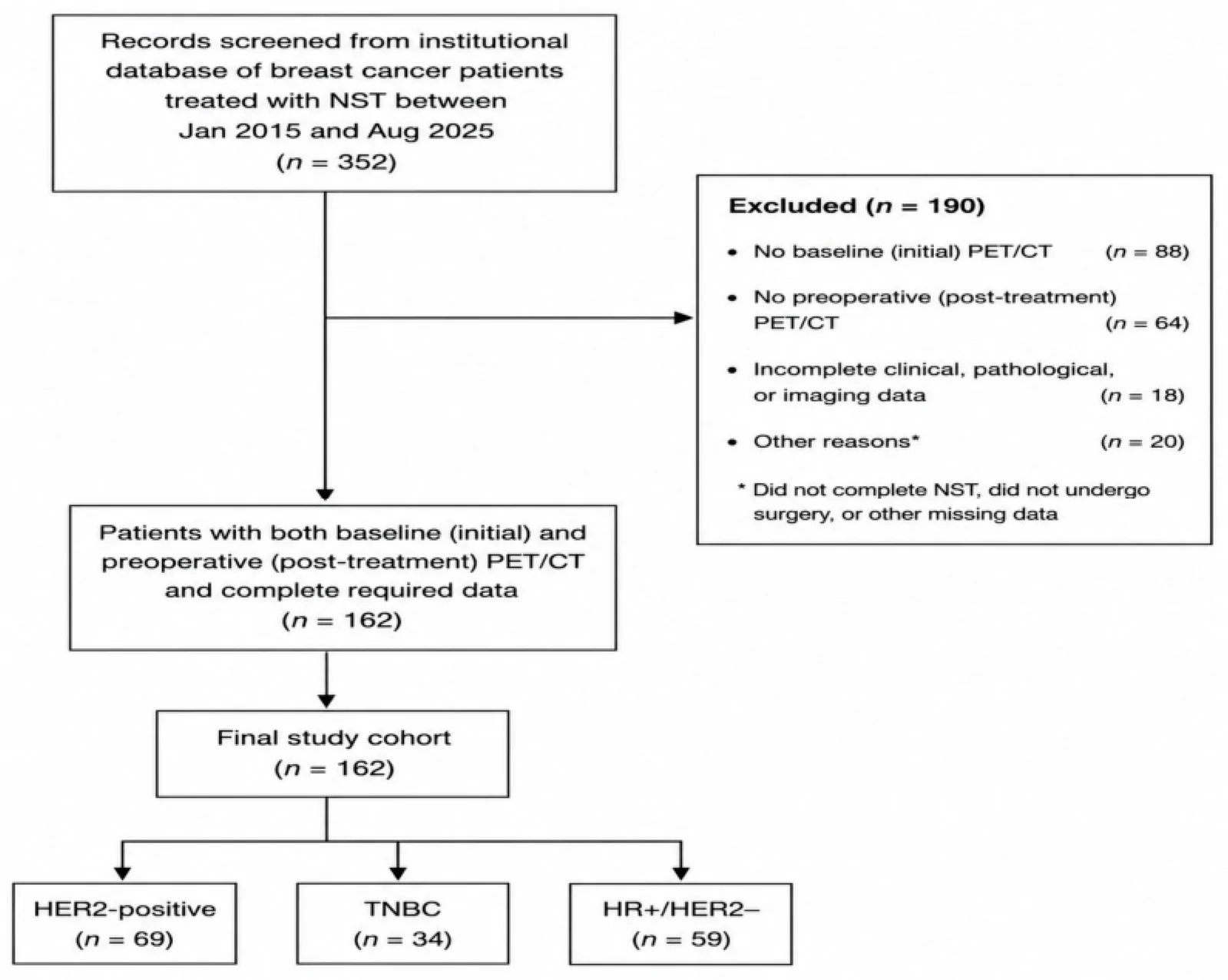
Flow of patients through this study according to the eligibility criteria.

Generative AI was used solely to assist in the graphical preparation of the study flow diagram (Figure 2), based entirely on data provided by the authors. The final figure was reviewed and verified by the authors.

### 2.7. Ethical Considerations

This study was conducted in compliance with the principles of the Declaration of Helsinki. Due to the retrospective design, the requirement for informed consent was waived. Ethical approval for this study was obtained from the Institutional Review Board (IRB) of Istanbul University–Cerrahpaşa, Cerrahpaşa Faculty of Medicine (Approval No.: 508/2025, dated 13 July 2025).

## 3. Results

### 3.1. Baseline Clinical, Demographic, and Treatment Characteristics

Of 352 patient records screened, 162 met the eligibility criteria and were included in the final study cohort (Figure 2). Among these patients, 63 (38.9%) achieved pCR, whereas 99 (61.1%) had residual disease following NST. Baseline clinicopathological characteristics are summarized in Table 1. Patients who achieved pCR were more likely to have lower clinical T stage (T1–2 vs. T3–4, *p* = 0.032) and molecular subtype distribution differed significantly between the pCR and non-pCR groups (*p* < 0.001), with higher pCR rates observed in TNBC and HER2-positive tumors. No significant differences were observed between the pCR and non-pCR groups regarding age, menopausal status, histological subtype, tumor grade, Ki-67 proliferation index, or clinical nodal stage.

Neoadjuvant treatment characteristics according to molecular subtype are presented in Supplementary Table S1. Most patients received anthracycline-based chemotherapy. Among patients with HER2-positive disease, 75.4% received dual anti-HER2 therapy, whereas platinum-containing chemotherapy was administered in 47.1% of patients with TNBC. Immunotherapy was used in 5.9% of patients with TNBC.

To further assess potential treatment-related differences in pathological response, major subtype-specific treatment components were compared according to pCR status (Supplementary Table S2). Among patients with HER2-positive disease, the use of dual anti-HER2 therapy did not differ significantly between patients with and without pCR (71.8% vs. 80.0%, respectively; *p* = 0.433). Similarly, among patients with TNBC, platinum use did not differ significantly according to pCR status (57.1% vs. 40.0%, respectively; *p* = 0.324).

### 3.2. Baseline and Post-Treatment PET/CT Characteristics

Baseline and post-treatment PET/CT parameters are presented in Table 2. Baseline primary tumor SUVmax and baseline axillary SUVmax did not differ significantly between patients who achieved pCR and those who did not. In contrast, post-treatment metabolic parameters showed significant associations with pCR. Patients achieving pCR had lower post-treatment primary tumor SUVmax (1.7 vs. 2.7, *p* < 0.001) and lower post-treatment axillary SUVmax (1.1 vs. 1.3, *p* = 0.011).

Similarly, treatment-induced metabolic changes were significantly greater among patients with pCR. Median ΔPrimary Tumor SUVmax was 82.8% in the pCR group compared with 65.4% in the non-pCR group (*p* < 0.001), while median ΔAxillary SUVmax was 84.7% and 74.6%, respectively (*p* < 0.001).

### 3.3. Predictors of Pathological Complete Response

The results of univariable and multivariable logistic regression analyses are summarized in Table 3. In univariable analysis, lower clinical T stage, HER2-positive and TNBC subtypes, lower post-treatment primary and axillary SUVmax values, and higher ΔPrimary Tumor SUVmax and ΔAxillary SUVmax were significantly associated with pCR, whereas baseline axillary SUVmax showed a borderline association.

Given the mathematical collinearity between baseline, post-treatment, and ΔSUVmax parameters, these variables were evaluated in separate multivariable models. Model 1 included ΔSUVmax parameters together with eligible clinicopathological covariates. In this model, HER2-positive subtype (OR 5.52, 95% CI 2.04–14.94, *p* < 0.001) and higher ΔPrimary Tumor SUVmax (OR 1.05, 95% CI 1.02–1.08, *p* < 0.001) were independently associated with pCR. ΔAxillary SUVmax was not independently associated with pCR (OR 1.00, 95% CI 0.97–1.02, *p* = 0.877).

Model 2 included baseline and post-treatment SUVmax parameters together with the same clinicopathological covariates. In this model, HER2-positive subtype (OR 5.21, 95% CI 2.12–12.78, *p* < 0.001), TNBC subtype (OR 4.31, 95% CI 1.44–12.93, *p* = 0.009), and lower post-treatment primary tumor SUVmax (OR 0.54, 95% CI 0.38–0.76, *p* < 0.001) were independently associated with pCR. Baseline axillary SUVmax showed a borderline association (OR 1.15, 95% CI 0.99–1.27, *p* = 0.066), whereas post-treatment axillary SUVmax was not independently associated with pCR (OR 0.92, 95% CI 0.72–1.18, *p* = 0.546).

The AUC was 0.836 (95% CI 0.775–0.898) for Model 1 and 0.849 (95% CI 0.786–0.909) for Model 2 (Supplementary Figure S1). Precision–recall analysis yielded an AUPRC of 0.739 for Model 1 and 0.741 for Model 2. Calibration plots comparing predicted and observed pCR probabilities for Model 1 and Model 2 are presented in Supplementary Figure S2. Bootstrap internal validation with 1000 resamples yielded optimism-corrected AUCs of 0.805 for Model 1 and 0.819 for Model 2.

The incremental value of PET-derived parameters beyond clinicopathological factors was additionally evaluated by comparison with clinical-only models including tumor grade, clinical T stage, and molecular subtype. In the corresponding complete-case populations, addition of ΔSUVmax parameters in Model 1 increased the AUC from 0.736 to 0.836 (ΔAUC = 0.101; DeLong *p* = 0.001), while the addition of PET parameters in Model 2 increased the AUC from 0.716 to 0.849 (ΔAUC = 0.132; DeLong *p* < 0.001). Likelihood-ratio tests were significant for both comparisons (χ^2^ = 24.012 and 33.888, respectively; both *p* < 0.001).

**Figure 3:**
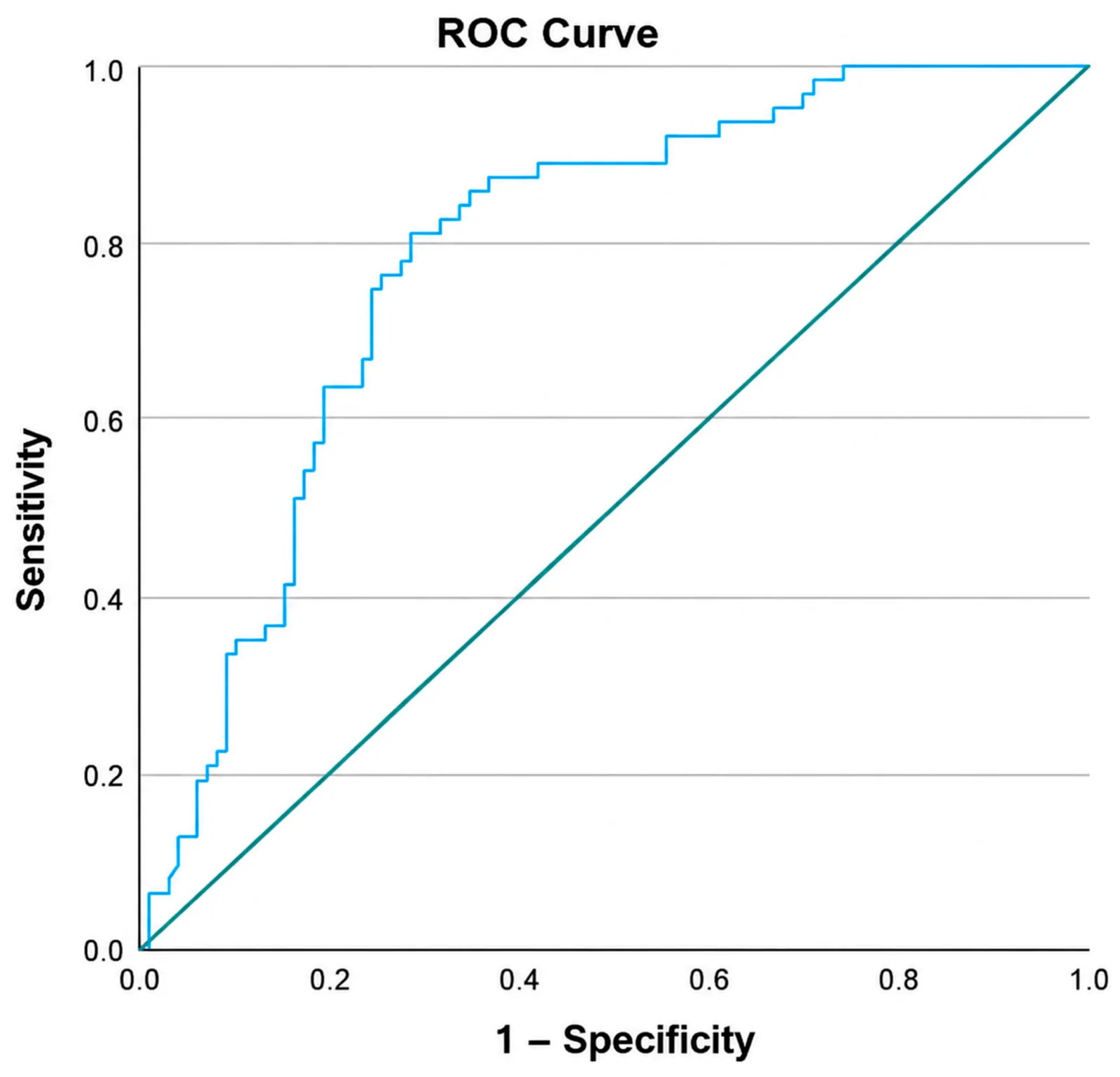
Receiver operating characteristic (ROC) curve demonstrating the discriminatory performance of ΔPrimary Tumor SUVmax for predicting pCR following NST. ΔPrimary Tumor SUVmax showed good predictive performance, with an area under the curve (AUC) of 0.787 (95% CI, 0.717–0.858). The diagonal line represents the line of no discrimination.

In the individual ROC analyses of the PET/CT-derived parameters, ΔPrimary Tumor SUVmax predicted pCR with an AUC of 0.787 (95% CI 0.717–0.858, *p* < 0.001). The exploratory cutoff derived using the Youden index was 72.2%, yielding a sensitivity of 87.3% and a specificity of 63.6% within the present cohort (Figure 3). Post-treatment primary tumor SUVmax demonstrated discriminatory performance for non-pCR, with an AUC of 0.769 (95% CI 0.697–0.840, *p* < 0.001). The exploratory cutoff of 2.67 yielded a sensitivity of 53.1% and a specificity of 90.5% within the present cohort (Supplementary Figure S3).

### 3.4. Subgroup Analysis According to Molecular Subtype

To investigate whether molecular subtype modified the predictive effect of metabolic response, interaction analyses were performed. No statistically significant interaction was observed between ΔPrimary Tumor SUVmax and molecular subtype (HER2-positive vs. HR+/HER2−: *p* = 0.282; TNBC vs. HR+/HER2−: *p* = 0.481).

Subgroup analyses according to molecular subtype are presented in Table 4. The association between ΔPrimary Tumor SUVmax and pCR was significant in the HER2-positive subgroup (OR 1.07, 95% CI 1.03–1.11, *p* < 0.001), whereas only a trend toward significance was observed in TNBC (OR 1.07, 95% CI 0.99–1.15, *p* = 0.075) and HR+/HER2− disease (OR 1.03, 95% CI 0.99–1.08, *p* = 0.096).

**Figure 4:**
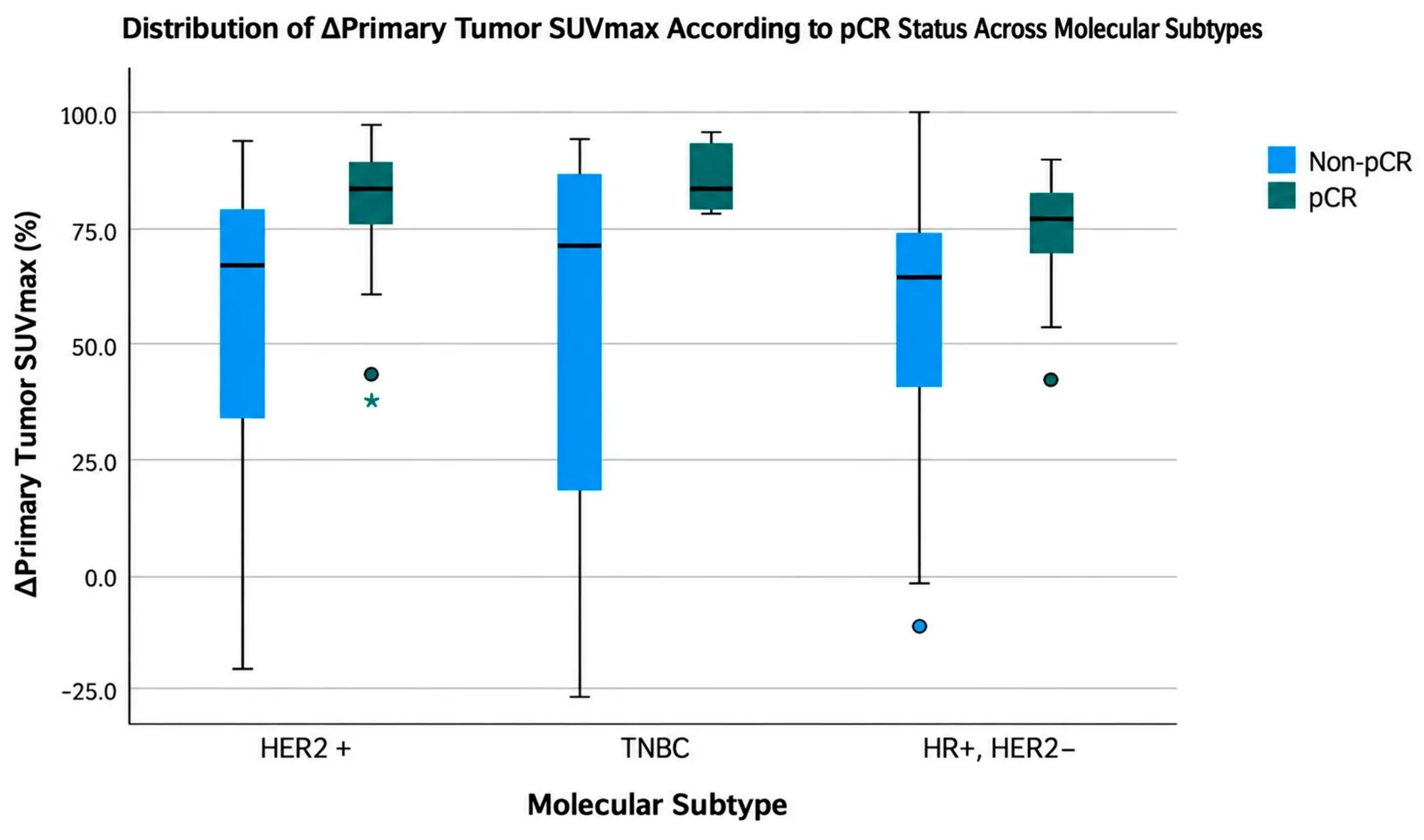
Box plots illustrating the distribution of ΔPrimary Tumor SUVmax in patients with and without pCR across molecular subtypes. The central line represents the median, boxes indicate the interquartile range, whiskers represent the minimum and maximum values excluding outliers, circles denote outliers and asterisks (*) denote extreme outliers.

The distribution of ΔPrimary Tumor SUVmax according to pCR status across molecular subtypes is shown in Figure 4. Across all molecular subtypes, patients achieving pCR tended to demonstrate greater reductions in primary tumor metabolic activity than those without pCR, with the most pronounced separation observed in the HER2-positive subgroup.

## 4. Discussion

In this retrospective study, we evaluated the association between PET/CT-derived metabolic parameters and pCR following NST. In separate multivariable models, lower post-treatment primary tumor SUVmax and greater ΔPrimary Tumor SUVmax were independently associated with pCR, whereas axillary PET/CT parameters did not provide independent predictive information. The addition of PET-derived parameters to clinical models including tumor grade, clinical T stage, and molecular subtype significantly increased model discrimination, suggesting incremental information beyond these clinicopathological factors. In exploratory subtype analyses, the association between ΔPrimary Tumor SUVmax and pCR reached statistical significance in the HER2-positive subgroup, although no statistically significant interaction with molecular subtype was observed.

Previous meta-analyses have demonstrated that PET/CT-derived metabolic response is associated with pCR following NST [7,8,13]. In the literature, baseline, post-treatment, and treatment-induced changes in PET parameters have all been investigated as potential predictors of pCR, with inconsistent results across studies [12]. In our cohort, baseline primary tumor SUVmax was not associated with pCR, whereas both post-treatment SUVmax and ΔPrimary Tumor SUVmax were significantly associated with pCR.

Our findings are consistent with those reported by Gelardi et al., who prospectively evaluated PET/CT response after neoadjuvant therapy and found that baseline metabolic parameters were not predictive of pCR, whereas post-treatment PET measurements were significantly associated with pCR [12]. Similarly, Kazerouni et al. performed serial FDG PET/CT examinations before treatment, during treatment, and before surgery and reported that baseline PET parameters were not associated with pCR, while treatment-induced metabolic changes were predictive of pCR [14]. However, the prognostic and predictive value of baseline PET parameters remains controversial. Several studies have reported an association between higher baseline SUVmax values and increased likelihood of achieving pCR [15,16,17], whereas others have failed to demonstrate such a relationship or have reported conflicting results [18,19,20]. These discrepancies may be related to differences in patient populations, molecular subtype distributions, treatment regimens, imaging protocols, and the PET-derived metrics evaluated. Taken together, the available evidence and our findings suggest that treatment-induced metabolic changes may provide additional information regarding treatment sensitivity beyond baseline metabolic activity alone. In addition to treatment-induced metabolic change, residual metabolic activity after completion of NST was independently associated with pCR in a separate multivariable model. In our cohort, ΔPrimary Tumor SUVmax demonstrated higher sensitivity, whereas post-treatment SUVmax showed higher specificity for predicting pCR based on the exploratory ROC-derived thresholds. These findings suggest that the magnitude of metabolic response and the degree of residual metabolic activity may provide complementary information regarding treatment response.

The incremental value analysis provided additional insight into the contribution of PET-derived parameters beyond clinicopathological factors. Compared with clinical-only models, the addition of PET-derived parameters resulted in ΔAUCs of 0.101 and 0.132 for Models 1 and 2, respectively, with significant DeLong and likelihood-ratio tests in both comparisons. These findings suggest that PET-derived parameters may provide additional discriminatory information when considered alongside established clinicopathological factors. Model performance was also evaluated using precision–recall analysis and calibration plots, while bootstrap internal validation resulted in some attenuation of the AUCs, with optimism-corrected values remaining above 0.80 for both models. Nevertheless, these findings reflect internal model performance within the present cohort and should therefore be interpreted cautiously until confirmed in independent external cohorts.

It should also be emphasized that PET/CT in the present study was performed after completion of NST and before surgery; therefore, our findings reflect post-treatment preoperative assessment rather than early prediction of treatment response. Although PET/CT-derived parameters may provide additional information regarding the likelihood of residual disease before surgery, our findings should not be interpreted as evidence for PET-guided modification in surgical or other clinical management. Nevertheless, the association between treatment-induced metabolic changes and pCR supports further investigation of whether metabolic changes assessed earlier during NST may have predictive value.

The impact of molecular subtype on the relationship between PET/CT-derived metabolic response and pCR remains an area of ongoing investigation. Several studies have suggested that the association between PET/CT parameters and pCR may vary across breast cancer subtypes [21,22,23], leading to increasing interest in subtype-specific response assessment and PET-guided treatment adaptation strategies [10,11,24]. This approach has been particularly explored in HER2-positive and triple-negative breast cancer, where early metabolic response may have implications for treatment escalation or de-escalation.

However, the available literature remains heterogeneous. Humbert et al. reported that the reduction in SUV after the first cycle of neoadjuvant chemotherapy was significantly greater in HER2-positive and triple-negative tumors than in luminal tumors and that ΔSUV was predictive of pCR only in HER2-positive disease [22]. In contrast, Groheux et al. demonstrated subtype-specific differences in the predictive value of PET parameters, reporting that changes in metabolic activity (ΔSUVmax and ΔTLG) were most strongly associated with pCR in TNBC and HR-positive/HER2-negative tumors, whereas post-treatment SUVmax showed superior predictive performance in HER2-positive disease [21]. Similarly, de Cremoux et al. reported that ΔSUVmax was significantly associated with pCR in patients with ER-positive/HER2-negative breast cancer [25].

In our study, interaction analysis did not demonstrate a statistically significant modification in the association between ΔPrimary Tumor SUVmax and pCR according to molecular subtype. In exploratory subtype analyses, the association reached statistical significance in the HER2-positive subgroup, but not in the TNBC or HR+/HER2− subgroups. However, the limited sample sizes and number of pCR events within individual subgroups should be considered when interpreting these findings. The discrepant findings across studies may be explained by differences in patient populations, treatment regimens, imaging timing, PET-derived parameters evaluated, and subtype distribution.

Compared with primary tumor metabolic response, data regarding the predictive value of axillary PET/CT parameters are relatively limited and the available results remain heterogeneous [26,27]. In our study, both post-treatment axillary SUVmax and ΔAxillary SUVmax were associated with pCR in univariable analyses; however, these associations did not remain significant in the corresponding multivariable models. In addition, our study evaluated overall pCR rather than axillary pathological response specifically, and this limitation should be considered when interpreting these findings, as it may have reduced our ability to detect associations between axillary metabolic response and nodal treatment outcomes.

This study has several limitations. Its retrospective design and single-center setting may have introduced selection bias and may limit the generalizability of the findings. The number of patients and pCR events within individual molecular subtypes, particularly in the TNBC and HR+/HER2− subgroups, was limited, which may have reduced the statistical power and robustness of the subgroup and interaction analyses. PET/CT assessments were available only at baseline and after completion of NST before surgery. Therefore, we were unable to evaluate the predictive value of interim metabolic response, which has been investigated in several PET-guided treatment adaptation studies. Treatment regimens were not completely homogeneous and reflected changes in real-world clinical practice over the long study period. Treatment selection was also strongly related to molecular subtype. Although the distributions of dual anti-HER2 therapy among patients with HER2-positive disease and platinum use among patients with TNBC did not differ significantly according to pCR status, the relatively small subtype-specific sample sizes and limited exposure to some treatments, particularly immunotherapy, precluded robust multivariable adjustment for individual treatment components. Therefore, residual treatment-related confounding cannot be excluded and should be considered when interpreting subtype-specific findings. Although axillary PET parameters were evaluated, axillary pathological complete response was not assessed separately, and analyses were based on overall pCR. Therefore, the predictive value of axillary metabolic response for nodal treatment outcomes may have been underestimated. PET/CT measurements were performed by a single nuclear medicine physician; therefore, inter-observer reliability could not be assessed. BMI data were also not available in this retrospective cohort and therefore could not be evaluated as a potential factor influencing SUV measurements. In addition, the ROC-derived SUVmax and ΔSUVmax thresholds were identified and evaluated within the same retrospective cohort and were not subjected to internal or external validation. Therefore, the observed sensitivity and specificity may represent optimistic estimates of performance. These cohort-derived thresholds should be considered exploratory and should not be interpreted as clinically validated cutoffs. Validation in independent prospective cohorts is required before their clinical application. Although bootstrap internal validation was performed for the multivariable models, external validation was not available; therefore, their performance and generalizability require confirmation in independent cohorts. Finally, SUVmax was used as the primary PET-derived parameter, whereas other volumetric PET metrics such as metabolic tumor volume (MTV) and total lesion glycolysis (TLG) were not available for analysis.

## 5. Conclusions

In this retrospective analysis, post-treatment primary tumor SUVmax and ΔPrimary Tumor SUVmax were associated with pCR in separate multivariable models, whereas baseline primary tumor SUVmax was not associated with pCR. No significant interaction between ΔPrimary Tumor SUVmax and molecular subtype was observed. In exploratory subtype analyses, the association reached statistical significance in the HER2-positive subgroup but not in the TNBC or HR+/HER2− subgroups; however, these findings should be interpreted in light of the limited sample sizes and number of pCR events within individual subgroups. Further prospective studies with larger subtype-specific cohorts and external validation are warranted.

## Figures and Tables

**Table 1:** Baseline Clinicopathological Characteristics According to Pathological Complete Response Status.

		Overall Cohort (*n* = 162)		Non-pCR (*n* = 99)		pCR (*n* = 63)		*p*
Age, *n* (%)	<50	84	52%	47	47%	37	59%	0.162
	≥50	78	48%	52	53%	26	41%	
Menopause status, *n* (%)	Premenopausal	90	56%	52	53%	38	60%	0.331
	Postmenopausal	72	44%	47	47%	25	40%	
Histological Subtype, *n* (%)	IDC	139	86%	81	82%	58	92%	0.069
	Other *	23	14%	18	18%	5	8%	
Grade, *n* (%)	2	73	45%	50	51%	23	37%	0.081
	3	89	55%	49	49%	40	63%	
Ki-67%, *n* (%)	<30	71	44%	46	46%	25	40%	0.396
	≥30	91	56%	53	54%	38	60%	
T stage, *n* (%)	T1–2	110	68%	61	62%	49	78%	0.032
	T3–4	52	32%	38	38%	14	22%	
N stage, *n* (%)	N0	15	9%	6	6%	9	14%	0.250
	N1	79	49%	48	48%	31	49%	
	N2	40	25%	25	25%	15	24%	
	N3	28	17%	20	20%	8	13%	
Subtype, *n* (%)	HER2+	69	43%	30	30%	39	62%	<0.001
	TNBC	34	21%	20	20%	14	22%	
	HR+/HER2−	59	36%	49	49%	10	16%	

Abbreviations: IDC, invasive ductal carcinoma; HER2, human epidermal growth factor receptor 2; TNBC, triple-negative breast cancer; HR, hormone receptor; pCR, pathologic complete response. * Other histological subtypes included invasive lobular carcinoma (ILC) and mixed invasive ductal/lobular carcinoma (IDC/ILC).

**Table 2:** Baseline and Post-Treatment PET/CT Parameters According to Pathological Complete Response.

	Overall Cohort (*n* = 162)	Non-pCR (*n* = 99)	pCR (*n* = 63)	*p*
Baseline primary tumor SUVmax	10.7 (2.2–54.1)	9.7 (2.2–54.1)	11.3 (2.4–35.9)	0.267
Baseline axillary SUVmax *	6.1 (0.6–39.3)	5.1 (0.6–39.3)	6.9 (0.6–34.2)	0.111
Post-treatment primary tumor SUVmax	2.2 (0.4–35.3)	2.7 (0.6–35.3)	1.7 (0.4–11.4)	<0.001
Post-treatment axillary SUVmax *	1.2 (0.3–20.5)	1.3 (0.3–20.5)	1.1 (0.5–8.7)	0.011
ΔPrimary tumor SUVmax (%)	74 (−25–98.7)	65.4 (−25–96.9)	82.8 (36.9–98.7)	<0.001
ΔAxillary SUVmax (%) *	79 (−35–98.5)	74.6 (−35–97.5)	84.7 (10–98.5)	<0.001

Abbreviations: SUVmax, maximum standardized uptake value. Data are presented as median (range) unless otherwise specified. Categorical variables are presented as *n* (%). * Axillary PET parameters were evaluated only in patients with baseline PET-positive axillary disease (*n* = 131; non-pCR, *n* = 79; pCR, *n* = 52). Patients with PET-negative but biopsy-proven axillary involvement were excluded.

**Table 3:** Univariate and multivariate logistic regression analysis of factors associated with pCR.

Variables		Univariable Analysis		Multivariable Model 1		Multivariable Model 2	
		OR (95%CI)	*p*	OR (95%CI)	*p*	OR (95%CI)	*p*
Age	<50	0.63 (0.33–1.20)	0.163				
	≥50						
Menopause status	Premenopausal	0.72 (0.38–1.38)	0.331				
	Postmenopausal						
Grade	2	1.77 (0.92–3.38)	0.082	1.11 (0.44–2.79)	0.812	1.34 (0.59–3.06)	0.477
	3						
T stage	T1–2 (RC)	0.45(0.22–0.94)	0.034	0.43 (0.17–1.08)	0.076	0.67 (0.29–1.58)	0.370
	T3–4						
N stage	N0–1	0.69 (0.36–1.31)	0.262				
	N2–3						
Subtype	HR+HER2− (RC)						
	HER2+	6.37 (2.78–14.61)	<0.001	5.52 (2.04–14.94)	<0.001	5.21 (2.12–12.78)	<0.001
	TNBC	3.43 (1.31–8.99)	0.012	2.62 (0.78–8.85)	0.119	4.31 (1.44–12.93)	0.009
Ki-67	<30	1.31 (0.69–2.50)	0.397				
	≥30						
Baseline primary tumor SUVmax		1.01 (0.97–1.04)	0.523	—			
Baseline axillary SUVmax		1.04 (0.99–1.09)	0.059	—		1.15 (0.99–1.27)	0.066
Post-treatment primary tumor SUVmax		0.51 (0.36–0.72)	<0.001	—		0.54 (0.38–0.76)	<0.001
Post-treatment axillary SUVmax		0.73 (0.56–0.96)	0.024	—		0.92 (0.72–1.18)	0.546
ΔPrimary tumor SUVmax (%)		1.06 (1.03–1.09)	<0.001	1.05 (1.02–1.08)	<0.001	—	
ΔAxillary SUVmax (%)		1.02 (1.01–1.04)	0.006	1.00 (0.97–1.02)	0.877	—	

Abbreviations: RC, reference category; pCR, pathological complete response; HER2, human epidermal growth factor receptor 2; TNBC, triple-negative breast cancer; SUVmax, maximum standardized uptake value. Model 1 included clinicopathological covariates together with ΔSUVmax parameters. Model 2 included the same clinicopathological covariates together with eligible baseline and post-treatment SUVmax parameters. Variables with *p* < 0.10 in univariable analysis were entered into the corresponding multivariable model. Dashes indicate variables not entered into that model. Separate multivariable logistic regression models were performed within each molecular subtype. Each model included ΔPrimary Tumor SUVmax, clinical T stage, and tumor grade.

**Table 4:** Association between ΔPrimary Tumor SUVmax and pCR according to molecular subtype.

Molecular Subtype	Adjusted OR * (95% CI)	*p*
HER2+ (*n* = 69)	1.07 (1.03–1.11)	<0.001
TNBC (*n* = 34)	1.07 (0.99–1.15)	0.075
HR+, HER2− (*n* = 59)	1.03 (0.99–1.08)	0.096

* Separate multivariable logistic regression models were performed within each molecular subtype. Each model included ΔPrimary Tumor SUVmax, clinical T stage, and tumor grade.

## Data Availability

The data that support the findings of this study are available on request from the corresponding author. The data are not publicly available due to privacy or ethical restrictions.

## Supplementary Materials

The following supporting information can be downloaded at: https://www.mdpi.com/article/10.3390/diagnostics16182909/s1. Table S1: Neoadjuvant treatment characteristics according to molecular subtype; Table S2: Major treatment components according to pathological response within molecular subtypes; Figure S1: Receiver Operating Characteristic Curves for the Multivariable Models Predicting Pathological Complete Response; Figure S2: Calibration Plots of the Multivariable Models for Predicting pCR; Figure S3: Receiver Operating Characteristic (ROC) Curve of Post-Treatment Primary Tumor SUVmax for Predicting Non-Pathological Complete Response.
