# Supplementary material for: Association of PET/CT-Derived Metabolic Parameters with Pathological Complete Response After Neoadjuvant Systemic Therapy in Breast Cancer: An Analysis Across Molecular Subtypes
Source: Diagnostics (Basel). 2026 Sep 9;16(18):2909. doi: 10.3390/diagnostics16182909 (PMC13605125; doi:10.3390/diagnostics16182909)
Supplement: Supplementary file 1 [file diagnostics-16-02909-s001.zip › diagnostics-4523741-supplementary.pdf]

Supplementary File

Table S1

Table S2

Figure S1

Figure S2

Figure S3

Table S1. Neoadjuvant treatment characteristics according to molecular subtype			
Treatment characteristic	HER2+ (n=69)	TNBC (n=34)	HR+ HER2- (n=59)
Anthracycline-based chemotherapy, n (%)	66 (95.7%)	32(94.1%)	57(96.6%)
Dual anti-HER2 therapy, n (%)	52(75.4%)	-	-
Platinum use, n (%)	2 (2.9%)	16 (47.1%)	-
Immunotherapy use, n (%)	-	2(5.9%)	-

Table S2. Major treatment components according to pathological response within molecular subtypes

HER2-positive cohort (n=69)		Non-pCR (n=30)		pCR (n=39)		p
Dual anti-HER2 therapy, n (%)	No	6	20%	11	28%	0.433
	Yes	24	80%	28	72%	

TNBC cohort (n=34)		Non-pCR (n=20)		pCR (n=14)		p
Platinum use, n (%)	No	12	60%	6	43%	0.324
	Yes	8	40%	8	57%	

Abbreviations: pCR, pathological complete response; HER2, human epidermal growth factor receptor 2; TNBC, triple-negative breast cancer. Dual anti-HER2 therapy was evaluated within the HER2-positive subgroup, whereas platinum use was evaluated within the TNBC subgroup.

Figure S1. Receiver Operating Characteristic Curves for the Multivariable Models Predicting Pathological Complete Response

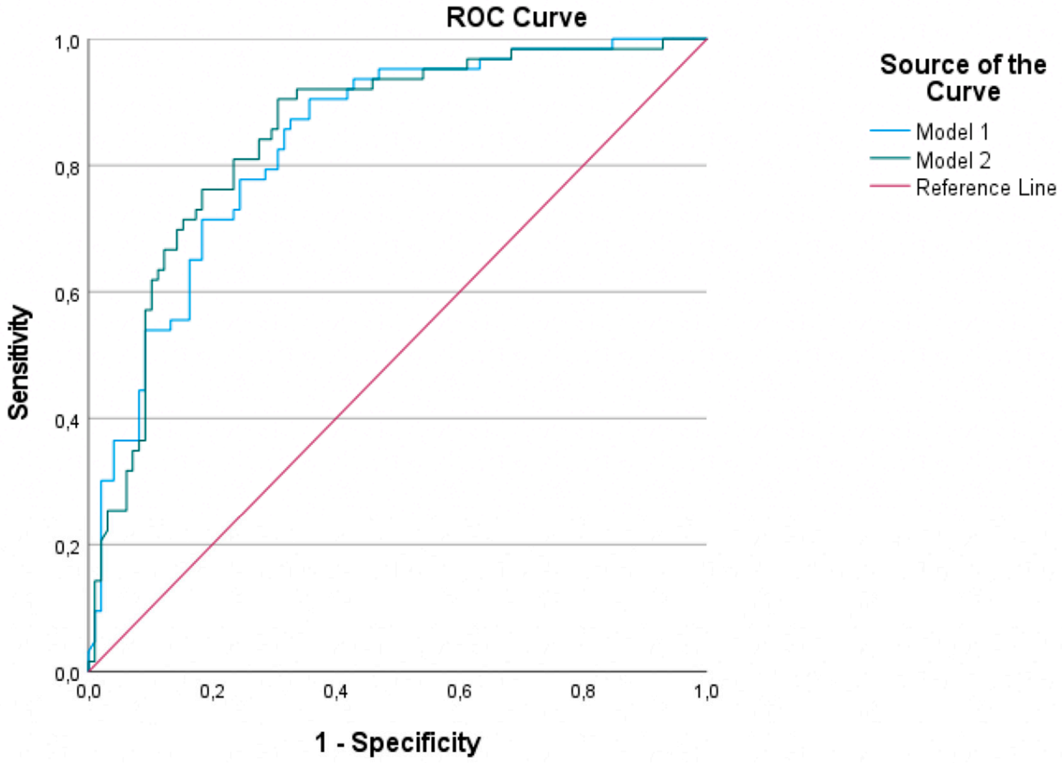

Figure S1. Receiver operating characteristic (ROC) curves showing the discriminatory performance of the two multivariable logistic regression models for predicting pathological complete response (pCR). Model 1, which included ΔSUVmax parameters together with eligible clinicopathological covariates, achieved an area under the curve (AUC) of 0.837 (95% CI, 0.775–0.898). Model 2, which included eligible baseline and post-treatment SUVmax parameters together with the same clinicopathological covariates, achieved an AUC of 0.848 (95% CI, 0.786–0.909).

Figure S2. Calibration Plots of the Multivariable Models for Predicting pCR

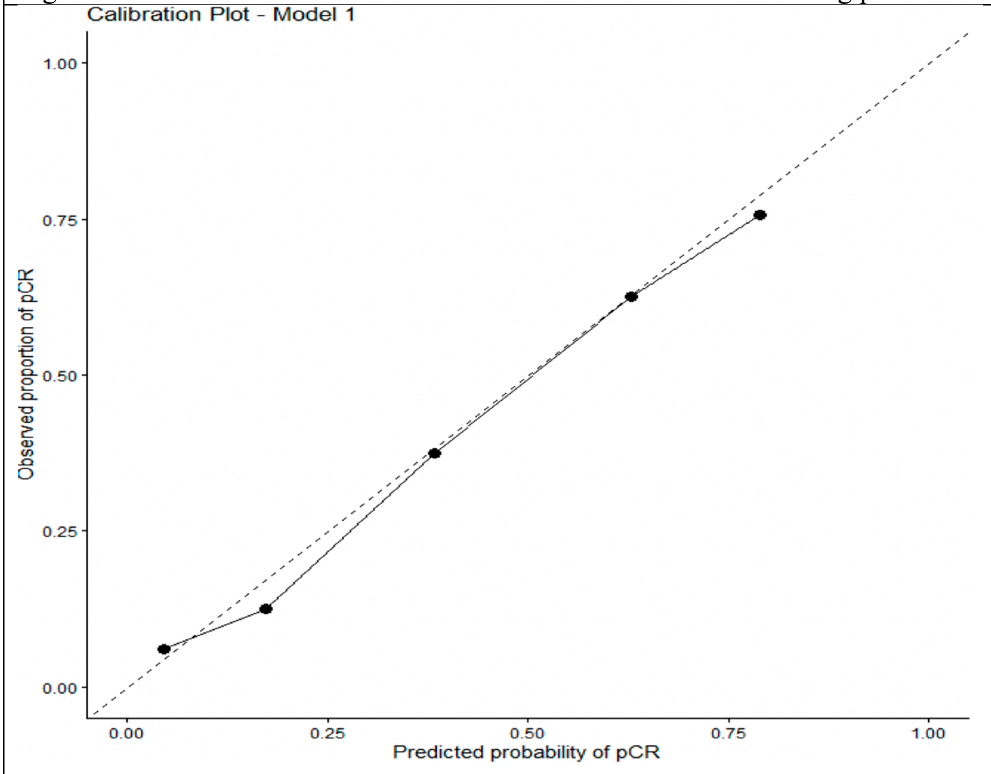

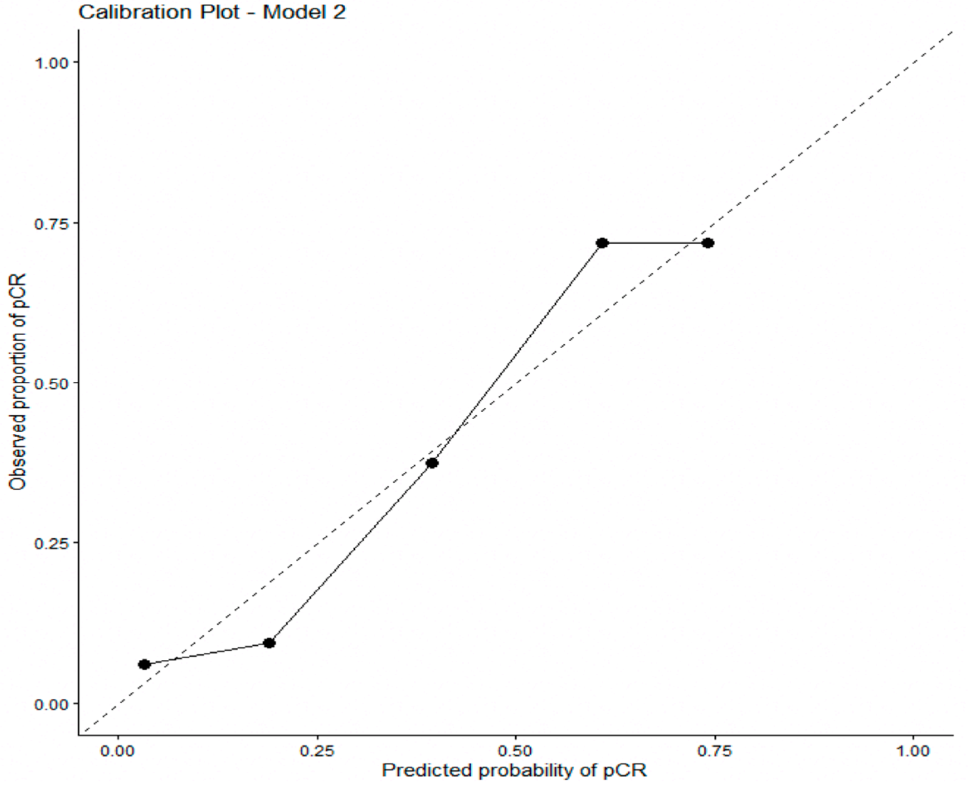

Figure S2. Calibration plots comparing predicted and observed probabilities of pCR for Model 1 and Model 2 . The dashed diagonal line represents perfect calibration.

Figure S3. Receiver Operating Characteristic (ROC) Curve of Post-Treatment Primary Tumor SUVmax for Predicting Non-Pathological Complete Response

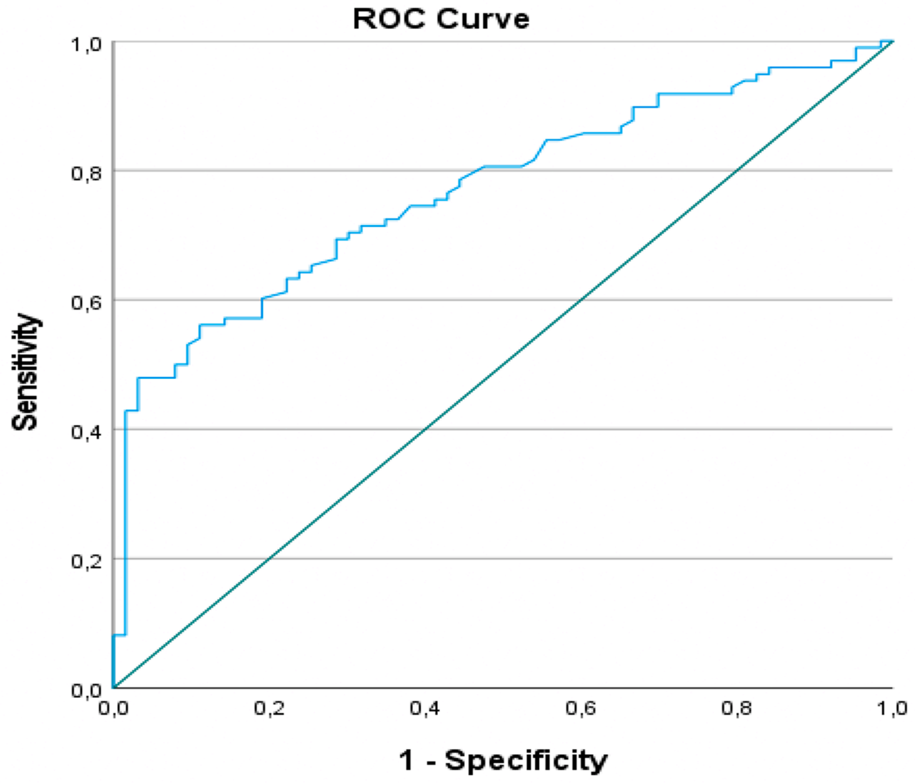

Figure S3. Receiver operating characteristic (ROC) curve demonstrating the discriminatory performance of post-treatment primary tumor SUVmax for predicting residual disease following neoadjuvant systemic therapy. The area under the curve (AUC) was 0.769 (95% CI, 0.697–0.840).
